# Mapping Kenyon cell inputs in *Drosophila* using dye electroporation

**DOI:** 10.1016/j.xpro.2023.102478

**Published:** 2023-10-20

**Authors:** Kaitlyn Elizabeth Ellis, Drue Marie Domagala, Sophie Jeanne Cecile Caron

**Affiliations:** 1 School of Biological Sciences, University of Utah, Salt Lake City, UT 84112, USA

**Keywords:** Cell Biology, Neuroscience

## Abstract

Here, we describe a technique for charting the inputs of individual Kenyon cells in the Drosophila brain. In this technique, a single Kenyon cell per brain hemisphere is photo-labeled to visualize its claw-like dendritic terminals; a dye-filled electrode is used to backfill the projection neuron connected to each claw. This process can be repeated in hundreds of brains to build a connectivity matrix. Statistical analyses of such a matrix can reveal connectivity patterns such as random input and biased connectivity.

For complete details on the use and execution of this protocol, please refer to Hayashi et al. (2022).^1^

## Highlights

• A technique to map mushroom body input connections in the adult *Drosophila* brain
• Photo-label an individual Kenyon cell to visualize its claw-shaped dendritic terminals
• Dye-fill the projection neuron connected to each claw-shaped dendritic terminal
• Construct a connectivity matrix to reveal and analyze global connectivity patterns

## Abstract (editor highlights)

Publisher’s note: Undertaking any experimental protocol requires adherence to local institutional guidelines for laboratory safety and ethics.

## Graphical abstract

**Figure:**
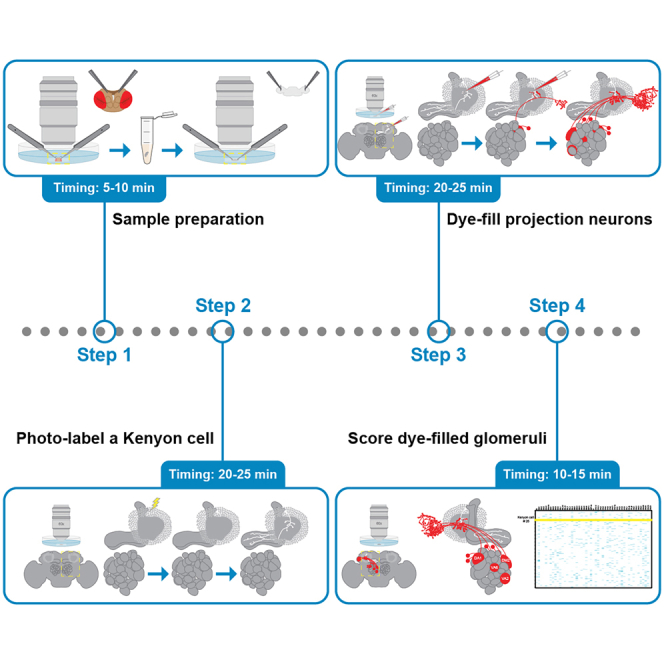

## Before you begin

### Preparation one: Generate the transgenic flies

**Timing: 2 weeks**

1. This protocol requires *D. melanogaster* flies that carry the transgenes necessary to express a photo-activatable form of the green fluorescent protein (PAGFP) in Kenyon cells. Any of the publicly available binary expression systems can be used to achieve that end. We use the GAL4/UAS system to do so, namely, the *nSynaptobrevin-GAL4* transgene, which is expressed in all neurons, and the *UAS-C3PAGFP* transgene.
2. Fly stocks and crosses are raised under standard conditions, using standard cornmeal agar medium in an incubator that is set to 25°C, 60% humidity and maintains a 12 h light/ 12 h dark cycle.
3. Adult flies are collected upon eclosion.

***Note:*** For high-quality photo-labeling results, dissect adult flies that are 1–3 days old.

***Note:*** This protocol can be used to map mushroom body connectivity in non-*melanogaster* species. The *nSynaptobrevin-GAL4* and *UAS-C3PAGFP* transgenes are currently available in *Drosophila sechellia* and *Drosophila simulans* and can be generated in other species.^2^^,^^3^^,^^4^

### Preparation two: Prepare the reagents

**Timing: 45 min**

4. Prepare the saline solution.
  a. Add the specified amount of the following chemicals to a 2 L single-use bottle: 12.62 g of NaCl, 0.75 g of KCl, 2.38 g of HEPES, 3.78 g of trehalose, 6.85 g of sucrose, 0.67 g of NaHCO_3_ and 0.24 g of NaH_2_PO_4_.
  b. Add 1,750 mL of deionized, filtered water to the same 2 L bottle and mix until the chemicals are dissolved.
  c. Add 4 mL of 1 M CaCl_2_ and 8 mL of 1 M MgCl_2_ to the solution and mix well.
  d. Add 310 μL of 10 M NaOH solution, one drop at a time, to the saline solution and mix after adding each drop.
  e. Add 238 mL of deionized, filtered water to the saline solution and mix well.
  f. Filter the resulting saline solution using a vacuum filtration system that has a polyesthersulfone membrane with a pore size of 0.22 mm (Corning).
  g. Use a pH meter to verify that the pH of the saline solution is approximately 7.3.
  h. Use an osmometer to verify that the osmolarity is within the optimal range: between 265 and 275 mOsm.
  i. Store at 4°C for up to one month.

***Note:*** We use disposable spatulas, weigh boats, and 2 L bottles to prevent soap residue from contaminating the saline solution. We refrain from using a magnetic stir bar for the same reason. Instead, we mix the saline solution with a disposable serological pipette.

***Note:*** The amount and the final concentration of each chemical is listed in a table in the materials and equipment section.

***Note:*** We use the FiveEasyPlus™ pH meter (Mettler Toledo) to measure pH and the VAPRO® Vapor Pressure Osmometer (Wescor®) to measure osmolarity.

***Note:*** The pH of the saline can be adjusted using NaOH (if it is too acidic <7.3) or HCl (if it is too basic >7.3). The osmolarity of the saline can be adjusted by adding trehalose (if it is too low <265 mOsm) or by adding Milli-Q water (if it is too high >275 mOsm) (See problem 1 in the troubleshooting section).

5. Prepare the collagenase.
  a. Reconstitute the collagenase using saline to a final concentration of 2 mg/mL.
  b. Store at −20°C for up to 1 year.
6. Prepare the fluorescent dye.
  a. Dilute 3000-Da Texas Red™ dextran dye using saline to a final concentration of 100 mg/mL.
  b. Store in the dark at −20°C.

### Preparation three: Prepare the electrodes

**Timing: 15–20 min**

7. Prepare electrodes by pulling borosilicate glass with filament.
  a. The parameters used to pull electrodes are listed in a table in the materials and equipment section.
  b. Refer to the maker’s guide for the micropipette puller for directions regarding general use, as instructions may vary between models.
8. Fire-polish the electrodes using a micro-forge to narrow their opening.
  a. Refer to the maker’s guide for the micro-forge for directions regarding general use, as instructions may vary between models.

***Note:*** We use filamented borosilicate glass that is 100 mm long, with an outside diameter of 1 mm and an inside diameter of 0.5 mm.

***Note:*** We use a P-2000 laser-based micropipette puller system (Sutter Instrument) to pull electrodes and a MF-900 microforge (Narishige) to fire polish the tip of each electrode.

## Key resources table

**Table:** 

REAGENT or RESOURCE	SOURCE	IDENTIFIER
**Chemicals, peptides, and recombinant proteins**		

CaCl_2_ solution (1 M in H_2_O)	Sigma-Aldrich	Cat#21115
Collagenase	Sigma-Aldrich	Cat#C5138
HEPES	Sigma-Aldrich	Cat#H3375
KCl	Sigma-Aldrich	Cat#P5405
MgCl_2_ solution (1 M in H_2_O)	Sigma-Aldrich	Cat#63069
NaCl	Sigma-Aldrich	Cat#S7653
NaHCO_3_	Sigma-Aldrich	Cat#S5761
NaH_2_PO_4_	Sigma-Aldrich	Cat#S5011
NaOH solution (10 M in H_2_O)	Sigma-Aldrich	Cat#72068
Sucrose	Sigma-Aldrich	Cat#S1888
SYLGARD® 184 silicon elastomer	Electron Microscopy Sciences	Cat#24236-10
Texas Red™ dextran dye	Thermo-Fisher	Cat#D3328
Trehalose	Sigma-Aldrich	Cat#T0167

**Deposited data**		

Raw data	This paper	https://github.com/ishanigan/hayashi-et-al-2022
Analyzed connectivity matrices	This paper	https://github.com/ishanigan/hayashi-et-al-2022

**Experimental models: Organisms/strains**		

*D. melanogaster: w*^*1118*^*;;;*	Bloomington Drosophila Stock Center	BDSC: 5905
*D. melanogaster: yw;10xUAS-IVSSyn21-mC3PA-GFP-p10*^*attP40*^*;;*	Aso et al.^11^	N/A
*D. melanogaster: w*^*1118*^*; GMR13F02-LexA*^*attP40*^*/CyO;;*	Bloomington Drosophila Stock Center	BDSC: 52460
*D. melanogaster: w∗;;Orco*^*2*^*;*	Bloomington Drosophila Stock Center	BDSC: 23130
*D. melanogaster: yw;N-Synaptobrevin-GAL4*^*2.1*^*;;*	Simpson Lab	N/A

**Software and algorithms**		

Code to analyze connectivity matrices	This paper	https://github.com/ishanigan/hayashi-et-al-2022
FIJI	Schindelin et al.^12^	https://imagej.nih.gov/ij/
Prairie View 5.4	Bruker	

**Other**		

1 L bottle top filter	Corning	431174
2 L bottle	Corning	
Controller	Sutter Instrument	MPC-200
Dovetail extension	Sutter Instrument	X285204
Dumont #55 forceps	Fine Science Tools	Cat#11295-51
Electrode holder	Warner Instruments	MP-515A
Filamented borosilicate glass	Sutter Instrument	Cat#BF100-50-10
Filter receiver and storage bottle	Thermo-Fisher	FB12566515
FiveEasyPlus™ pH meter	Mettler Toledo	FEP20
GaAsP detector	Hamamatsu Photonics	N/A
Inorganic membrane filter Anotop™ 10	GE Life Sciences, Whatman™	CA#6809-1022
MicroFil™	World Precision Instruments	MF34G-5
Micro forge	Narishige	Cat#MF-900
Micromanupulator	Sutter Instrument	Cat#MP-265
Micropipette puller	Sutter Instrument	RRID: SCR_018640
Mounting adapter plate	Sutter Instrument	X285210
Petri dish, 10 mm	Thermo-Fisher	Cat#FB0875711YZ
Pipette storage box	Sutter Instrument	Cat#BX20
PMT detector	Bruker	N/A
Pockels cell	Conotopics	Cat#350-80LA/BK-02
Rod holder assembly	Sutter Instrument	MP-ROD
Rotary optical encoder input device	Sutter Instrument	ROE-200
Stand	Sutter Instrument	MT-75
Tungsten 99.95% CS wire	California Fine Wire Company	Cat#100211
Ultima Investigator multiphoton microscope	Bruker	RRID: SCR_019807
Ultrafast Chameleon Ti:sapphire laser	Coherent	N/A
VAPRO® vapor pressure osmometer	Wescor®	
Water immersion objective lens, 60×	Olympus	Cat#N2667800

## Materials and equipment

**Table:** Saline solution

Reagent	Final concentration	Amount
NaCl	108 mM	12.62 g
KCl	5 mM	0.75 g
HEPES	5 mM	2.38 g
Trehalose	5 mM	3.78 g
Sucrose	10 mM	6.85 g
NaHCO_3_	4 mM	0.67 g
NaH_2_PO_4_	1 mM	0.24 g
MgCl_2_	4 mM	8 mL
CaCl_2_	2 mM	4 mL
ddH2O	N/A	Up to 2 L

**Table:** Parameters for pulling electrodes

Parameter	Value
Delay	200
Filament	4
Heat	340
Number of lines (cycles)	1
Number of loops	4
Time of last loop	5.4–5.85 s
Velocity	30

***Note:*** The parameters that we use to pull electrodes are optimized for the P-2000 laser-based micropipette puller system and filamented borosilicate glass pipettes (Sutter Instrument).

***Note:*** We use a program that is comprised of the parameters: delay, filament, heat, and velocity. The delay parameter dictates the timing of the start of the hard pull relative to the deactivation of the laser (we use a delay of 200, which means that the hard pull occurs after the deactivation of the laser). The filament parameter controls the scanning pattern of the laser (we use a filament of 4, which means that the scan length of the laser is set at 6.5 mm). The heat parameter controls the output power of the laser. The velocity parameter determines the velocity at which the puller bar must be moving before the hard pull occurs.

***Note:*** The values for the number of loops and the time of the last loop are displayed on the screen of the micropipette puller system after the hard pull occurs. Generally, the program will loop 4 times before a hard pull occurs. The heat value that we use in our program usually melts the glass enough to trip a hard pull within a timeframe of 5.4–5.85 s. Adjust the heat parameter accordingly if the hard pull occurs outside of this timeframe.

***Note:*** The resulting electrode should have a tip diameter of 1–2 μm.

**Table:** Parameters for imaging

Parameter	Live scan / image acquisition	Photo-label
Mode	Live scan	Single image
Objective lens	60×	60×
Image resolution	512 × 512	512 × 512
Pixel size	0.39 μm	0.39 μm
Pixel dwell time	4 μs	4 μs
Average frame	1 or 2	8
Laser wavelength	925 nm	710 nm
Laser power	1–14 mW	5–30 mW

***Note:*** The laser power is measured by first removing the objective lens from the turret and then placing the sensor directly below the objective lens socket.

***Note:*** An ‘average frame’ of one is used during live scan and dye electroporation. An ‘average frame’ of two is used during image acquisition.

**Table:** Parameters for dye electroporation

Parameter	Value
Train mode/type of pulse	Single (not repeat)
Train rate	N/A
Duration	0.5 ms
Delay	None
Voltage	10–50 V
Laser wavelength	925 nm
Laser power	1–14 mW
Number of pulses	1–3

## Step-by-step method details

***Note:*** A similar protocol was used in different studies.^1^^,^^4^^,^^5^

### Step one: prepare the *Drosophila* brain sample

**Timing: 5–10 min**

The purpose of this step is to prepare the brain such that the mushroom bodies are accessible for image acquisition and dye electroporation.

**Figure 1:**
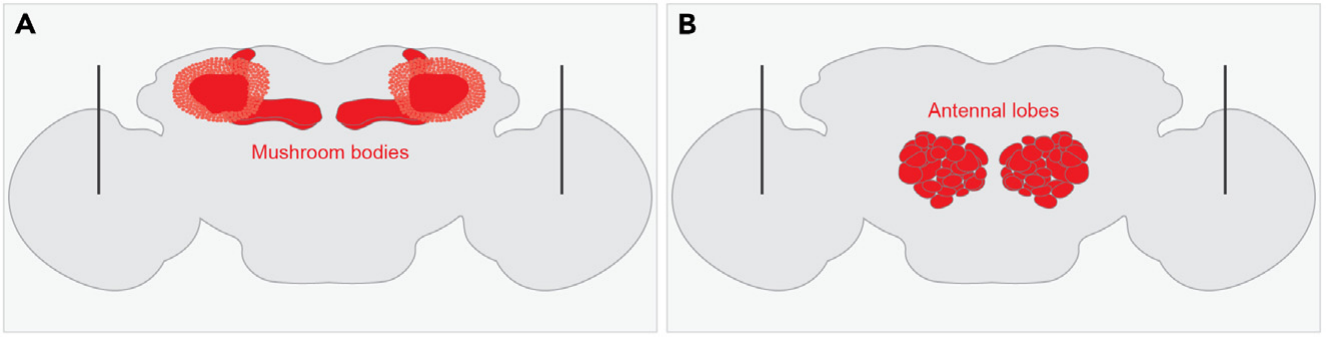
Pinning the brain (A) Schematic of the brain pinned with the posterior side facing upwards (and the anterior side facing the piece of SYLGARD®), the orientation where the mushroom body calyces are visible (colored red). This is the orientation used in step 2: photo-label a Kenyon cell and step 3: dye-fill projection neurons. (B) Schematic of the brain pinned with the anterior side facing upwards (and the posterior side facing the piece of SYLGARD®), the orientation where the antennal lobes are visible (colored red). This is the orientation used in step 4: score the dye-filled glomeruli.
  b. Create pins by cutting tungsten wire into small segments.
  c. Secure the brain by pinning through both optic lobes, into the SYLGARD®. Before proceeding to the steps that require two-photon microscopy, make sure that the pins are anchored and that both hemispheres of the brain are level.

1. Prepare two dishes, one in which to dissect the brain and another to hold the sample.
  a. Procure a small, 10 mm petri dish and line both the lid and the base with a thin piece of SYLGARD®, a material that will prevent the brain from adhering to the dish.
  b. Use the base of the petri dish as a container in which to dissect and the lid as a receptacle for the fully prepped sample.
  c. Fill both halves of the petri dish with saline solution.
2. Dissect the brain.^6^
  a. Anesthetize the fly; CO_2_ is suitable for this protocol.
  b. Use forceps to remove the head from the body, separate the cuticle from the brain, and remove as much trachea from the brain as possible.
3. Remove the thin, outermost layer of the brain to permit an electrode to pierce through the brain.
  a. Use a glass Pasteur pipette to transfer the brain from the bottom half of the petri dish to an aliquot of 2 mg/mL collagenase solution. Make sure to pre-wet the pipette to prevent the brain from sticking to the wall during transfer.
  b. Incubate the brain in the collagenase solution for approximately 30 s.
  c. Transfer the collagenase-treated brain directly to the lid of the petri dish.
4. Prepare the sample for two-photon microscopy.
  a. Orient the brain with the anterior side facing the piece of SYLGARD® and the posterior side facing upwards; this orientation will provide easy access to the calyx of the mushroom body and the somata of the Kenyon cells (Figure 1A).

### Step two: Photo-label a Kenyon cell

**Timing: 20–25 min**

The purpose of this step is to photo-label a Kenyon cell to visualize its claw-shaped dendritic terminals.

5. Prepare for two-photon microscopy.
  a. Situate the petri dish containing the brain sample under the objective lens with the dorsal side of the brain facing the micromanipulator.
  b. Switch to the 60× objective (if the sample was located using a lower objective) before using two-photon microscopy to visualize the brain.

***Note:*** We used an Ultima two-photon microscope (Bruker) equipped with an ultrafast Chameleon Ti: Sapphire laser (Coherent) modulated by a Pockels Cell (Conotopics) for both photo-labeling and image acquisition. The exact instrumental configuration described here may be different from instruments made by other manufacturers. For the imaging software, we used Prairie View 5.4 (Bruker).

***Note:*** The orientation of the brain for two-photon microscopy during steps 2 and 3 is dictated by the handedness of the micromanipulator system that is being used. We use a setup in which the micromanipulator is located to the left of the microscope, and, therefore, we situate the petri dish such that the dorsal side of the brain is facing left. Aligning the dorsal side of the brain with the micromanipulator is important, as this orientation will permit the dye-filled electrode used in step 3 to access the mushroom body calyces.

**Figure 2:**
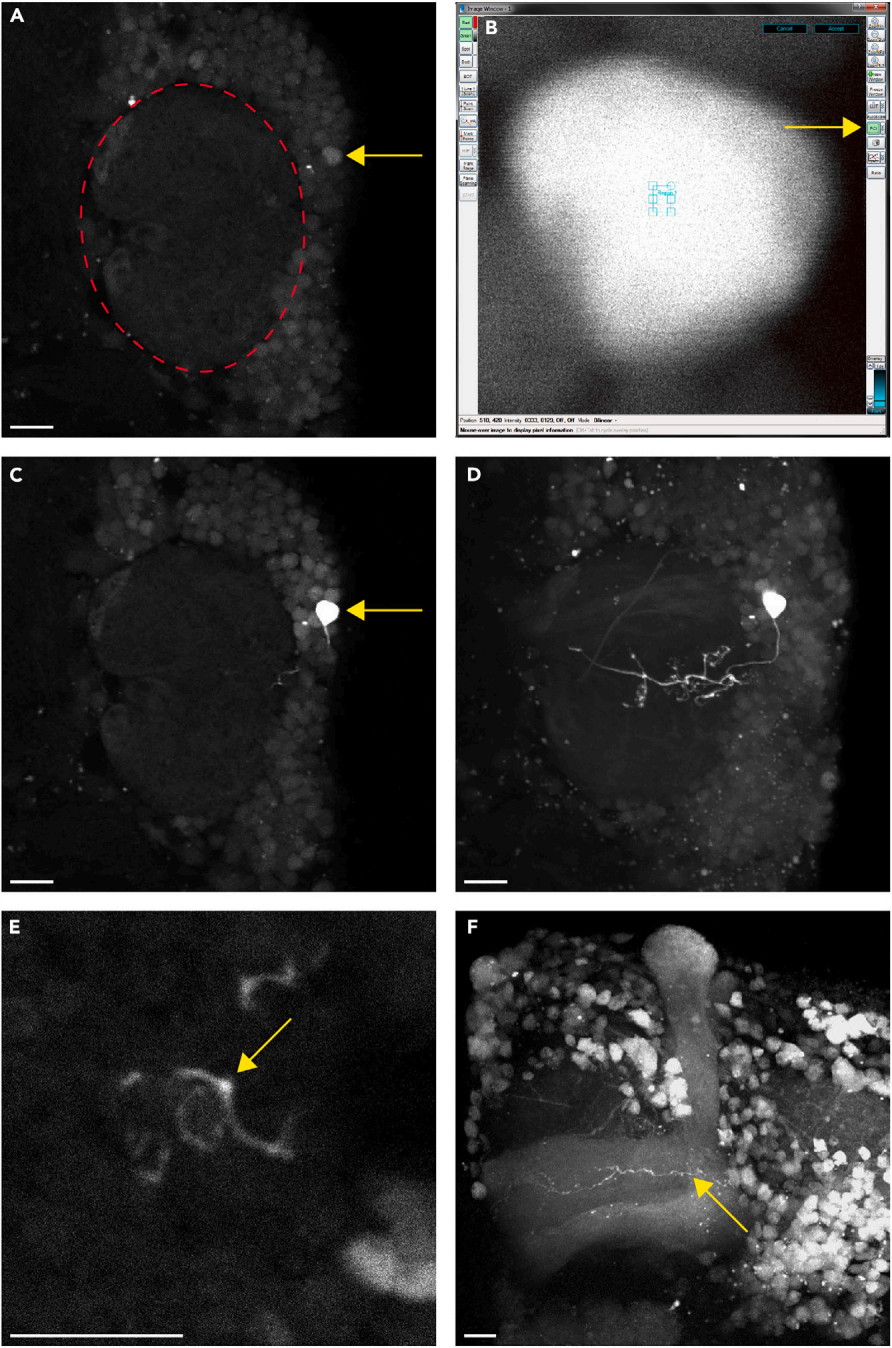
Photo-labeling a Kenyon cell (A) Locate the mushroom body calyx (outlined in red) and select a Kenyon cell soma (yellow arrow) to photo-label (digital zoom: 2). (B) Zoom in on the Kenyon cell soma, draw a small ROI (cyan box) within the boundaries of the soma, and photo-label the ROI as described in step 2: photo-label a Kenyon cell, sub-step 7 (digital zoom: 32). (C) The soma of the targeted Kenyon cell (yellow arrow) is brighter after photo-conversion (digital zoom: 2). (D) The dendritic arbors and claw-shaped terminals of the photo-labeled Kenyon cell are visible 10 min after photo-conversion (digital zoom: 2). (E) An enlarged view of one of the claws (yellow arrow) formed by the photo-labeled Kenyon cell (digital zoom: 8). (F) The axons of the photo-labeled Kenyon cell (yellow arrow) project in the gamma lobe of the mushroom body (imaged during step 4: score the dye-filled glomeruli) (digital zoom: 1.5). All images are composites, and all scale bars are 10 μm.
  d. Choose the Kenyon cell soma to be photo-labeled; zoom in to the point where it occupies most of the frame (suggested digital zoom factor: 32×) (Figure 2B).
  e. Stop the live scan mode.
7. Photo-convert the PA-GFP expressed by the selected Kenyon cell.
  a. Draw a region of interest (ROI) within the boundaries of the soma (Figure 2B).
  b. Configure the imaging software according to the photo-labeling parameters listed in the materials and equipment section.
  c. Tune the laser to 710 nm. Use the single image mode and collect 3 to 5 scans to induce the photo-conversion of PA-GFP.
  d. Tune the laser back to 925 nm, exit the ROI, and reset the digital zoom.
  e. In live scan mode, verify that only one Kenyon cell soma was photo-labeled during the process (Figure 2C) (See problem 2 in the troubleshooting section).
8. Wait for 10 min.
9. In live scan mode, view the calyx to confirm that the Kenyon cell was successfully photo-labeled. At this point, the entire morphology of the Kenyon cell should be noticeably brighter than the rest of the brain (Figure 2D).
10. Image the photo-labeled Kenyon cell.
  a. Set up a Z-series that encompasses the entire volume of the calyx (suggested digital zoom: 2×).
  b. Document the number of claw-shaped dendritic terminals of the photo-labeled Kenyon cell.

***Note:*** We used 1 μm steps for all Z-series taken during this protocol.

***Note:*** This image sequence of the calyx and photo-labeled Kenyon cell can serve as a guide for locating the claws during step 3.

6. Select a Kenyon cell to photo-label.
  a. Configure the imaging software according to the live scan parameters that are listed in the materials and equipment section.
  b. Tune the laser to 925 nm and use the live scan mode to visualize the sample.
  c. Locate the mushroom body calyx and the Kenyon cells; the somata of Kenyon cells are proximal to the calyx and have a spherical shape (Figure 2A).

***Optional:*** This procedure can also be performed on one Kenyon cell of the opposite hemisphere. This measure will increase the probability of successfully photo-labeling a Kenyon cell or, possibly, two (one per hemisphere).

### Step three: Dye-fill the projection neurons

**Timing: 20–25 min**

The purpose of this step is to use dye electroporation to dye-fill the projection neurons connected to the claw-shaped dendritic terminals — or 'claws' — of the photo-labeled Kenyon cell.

11. Prepare for dye electroporation.
  a. Keep the brain in the same orientation as step 2 (Figure 1A).
  b. Submerge the non-pulled end of one of the electrodes that was pulled in preparation step 3 in the aliquot of Texas Red™ dextran dye that was made in preparation step 2, sub-step 6. Allow the internal capillary to fill.
  c. Use a syringe, equipped with an inorganic membrane filter (GE Life Sciences Whatman™) and a MicroFil™ (World Precision Instruments) tip, to flush the electrode with saline and eliminate any bubbles that might obstruct its tip.
  d. Secure the electrode within its dedicated holder (Warner Instruments), which is located on the head stage of the micromanipulator (Sutter Instrument).
  e. Align the electrode with the brain.
  f. Subsequently, align the electrode with the calyx.

***Note:*** We use the MPC-365 Multi Motorized Narrow-Format Micromanipulator Control System (Sutter Instrument) to move the dye-filled electrode. This system is comprised of the MPC-200 controller, ROE-200, MP-265 narrow format stepper motor manipulator, and 4″ dovetail extension. To customize the MP-265 manipulator so it accommodates the electrodes we use for dye electroporation, we attach an MP-ROD rod holder (Sutter Instrument) to the manipulator and fit an MP-515A electrode holder (Warner Instruments) within the MP-ROD holder. We use an X28510 mounting adapter plate (Sutter Instrument) to affix the micromanipulator to an MT-75 stand (Sutter Instrument), which we place to the left of our microscope.

***Note:*** The movement of the manipulator is controlled via rotating knobs on a rotary optical encoder input device (ROE). Each ROE has modes that dictate the speed and fineness of the movement of the manipulator. We routinely use mode 0 (accelerated mode) on the ROE-200 (Sutter Instrument) to initially align the electrode with the brain and mode 1 to align it with the mushroom body calyx (under bright field microscopy). We use mode 4 (increased sensitivity and decreased speed) to control the electrode during the dye electroporation procedure (under two-photon microscopy).

**Figure 3:**
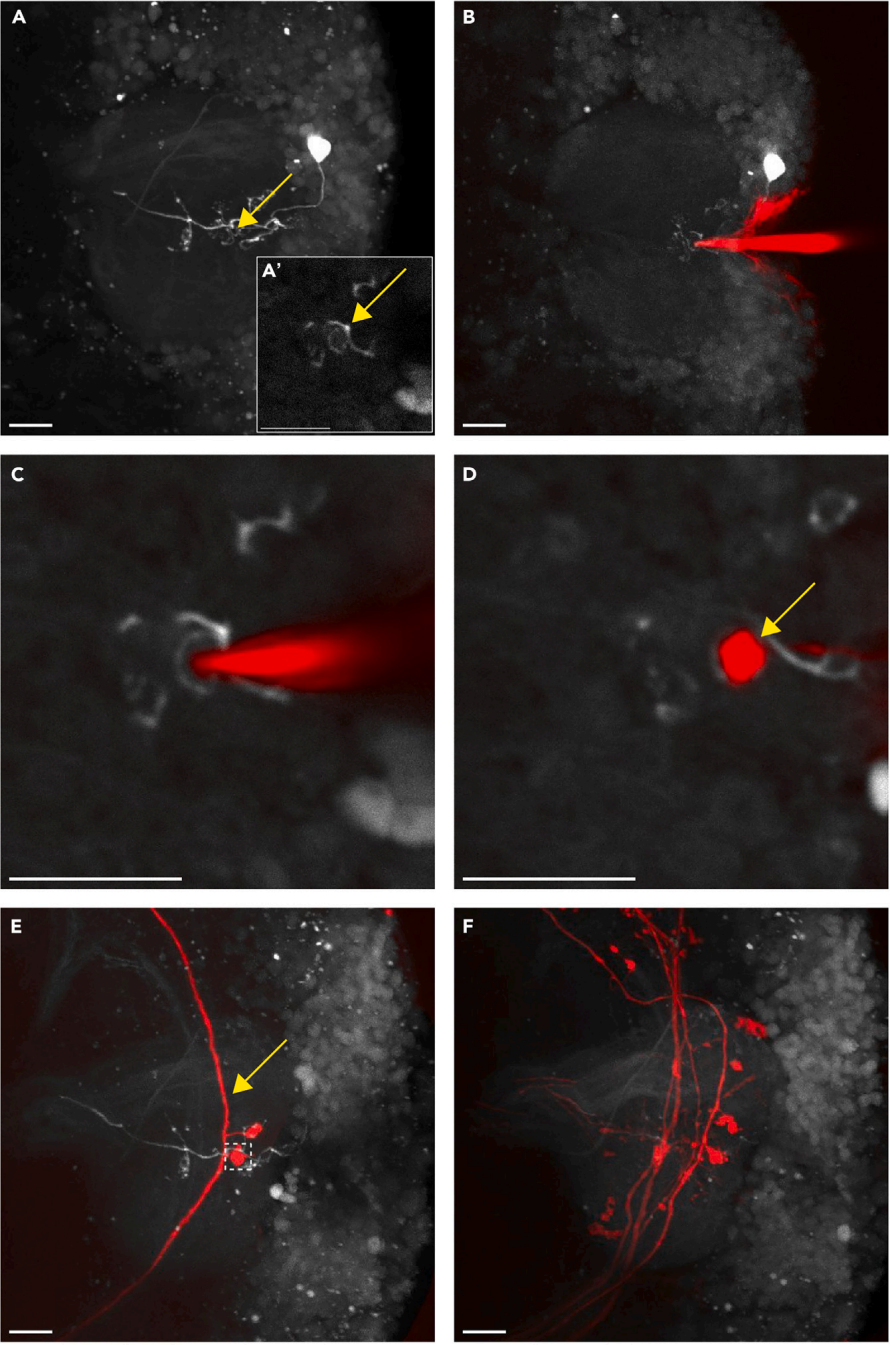
Dye-filling projection neurons (A) Align the dye-filled electrode with the mushroom body calyx (digital zoom: 2). (A′) Select a first claw to fill (yellow arrow) (digital zoom: 8). (B) Insert the electrode into the calyx, targeting the claw of interest. Note that the invagination that is created when the electrode is pushed into the calyx will not impact the tissue and the dye-labeling in any meaningful way (digital zoom: 2). (C) Verify that the electrode is located within the claw before electroporating dye into it (digital zoom: 8). (D) The presynaptic bouton (yellow arrow) of the dye-filled projection neuron is visible after dye electroporation and once the electrode is removed (digital zoom: 8). (E) Verify that only one projection neuron was dye-filled after the first electroporation. Only one axon should be visible (yellow arrow). Note that once the dye diffuses throughout the projection neuron, not only will the bouton that was initially filled (white box) be labeled, but the other boutons (in this case only 1 bouton) of that projection neuron will be visible as well (digital zoom: 2). (F) Fill as many claws as possible; in this case, five claws were filled leading to the dye-labeling of five projection neurons (digital zoom: 2). All images are composites, and all scale bars are 10 μm.
  e. Carefully situate the tip of the electrode into the center of the claw. To prevent off-target labeling, make sure that the tip of the electrode is located within the claw in all planes in which the claw is visible (Figures 3B and 3C).
  f. Manually administer one or two 10–50 V pulses to electroporate the dye into the presynaptic bouton formed by the projection neuron connected to the claw; the dye should quickly diffuse from the bouton to the main axon cable of the projection neuron (Figures 3D and 3E).
  g. Verify that only one projection neuron was filled with dye before proceeding to the next claw (Figure 3E) (See problem 4 in the troubleshooting section).
  h. Repeat sub steps 12d-g to fill as many of the claws as possible (Figure 3F).

***Note:*** We used the S88 Dual Output Square Pulse Stimulator (GRASS®) for dye electroporation.

12. Dye-fill the claws of the photo-labeled Kenyon cell.
  a. Configure the electrophysiology rig parameters for dye electroporation as specified in the materials and equipment section.
  b. Configure the imaging software according to the live scan parameters that are listed in the materials and equipment section.
  c. Verify that the laser is tuned to 925 nm.
  d. Using the live scan mode, locate, and select, the first claw of the photo-labeled Kenyon cell to fill with dye (Figure 3A).

13. Set up a Z-series and image the entire volume of the calyx, documenting the Kenyon cell and its dye-filled presynaptic partners (suggested digital zoom: 2×).

***Note:*** In most cases, it is easier to begin with the claw that is the most proximal to the surface of the calyx and is the most dorsally located. Larger claws are also easier to fill. It must also be noted that the claws that are located deep into the calyx, immediately above the projection neuron tracts, are the most difficult to fill, as off-target labeling is more likely (see problem 4 in the troubleshooting section).

***Note:*** While the suggested range of amplitudes for the pulses is 10–50 V, successful results have consistently been achieved with 30 V.

***Note:*** During the process of dye-filling projection neurons, Kenyon cells can also be labeled. While such labeling is not aesthetically pleasing, it will not affect the data.

### Step four: Score the dye-filled glomeruli

**Timing: 10–15 min**

The purpose of this step is to identify the olfactory glomeruli from which the dye-filled projection neurons originate.

14. Reverse the orientation of the brain to permit the visualization of the antennal lobes and mushroom body lobes.
  a. Return to the dissection scope that was used in step 1.
  b. Carefully remove the pins from the optic lobes.
  c. Flip the brain so the posterior side is facing the SYLGARD® and the anterior side is facing the objective lens (Figure 1B).
  d. Re-pin the brain and return to the two-photon microscope.
15. Image the antennal lobe.
  a. Configure the imaging software according to the live scan parameters for imaging the brain post-dye labeling that are listed in the materials and equipment section.
  b. Verify that the laser is tuned to 925 nm.
  c. Capture a Z-series that encompasses the entire antennal lobe and the somata of the projection neurons, which are located in three clusters surrounding the antennal lobe (the anterior-dorsal, lateral and ventral clusters).

**Figure 4:**
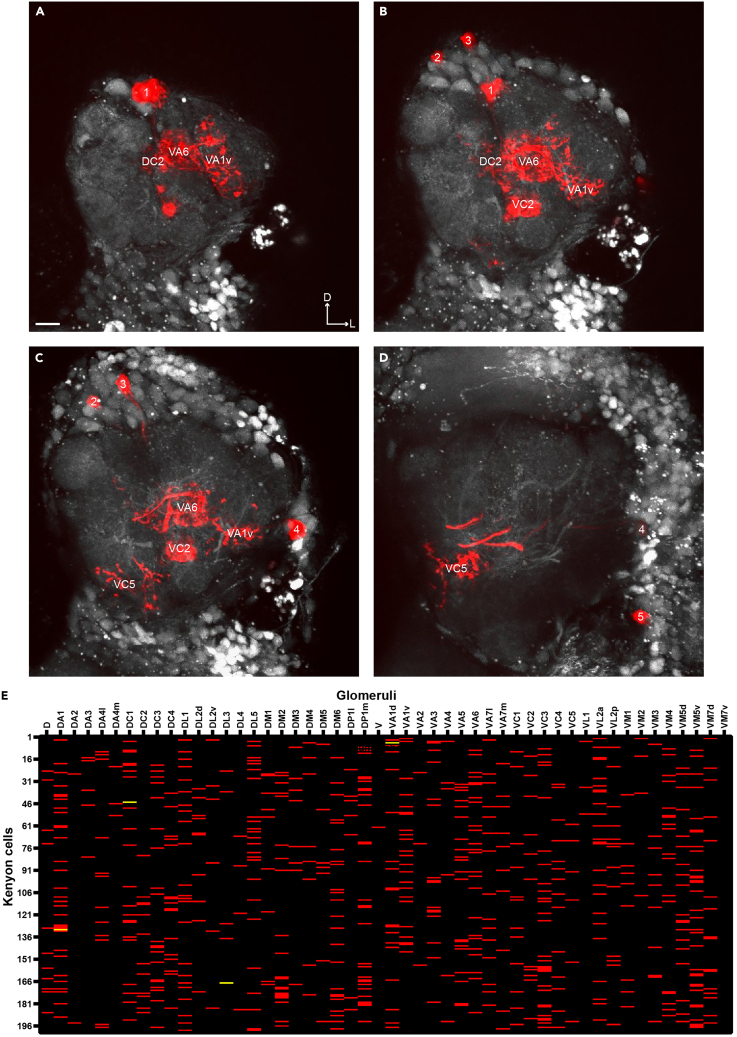
Scoring dye-filled glomeruli (A–D) Maximum intensity projections of four different planes in the antennal lobe comprising dye-filled glomeruli (the DC2, VA6, VA1v, VC2 and VC5) and projection neuron somata (1–5), arranged from the most anterior (panel A) to most posterior (panel D) (digital zoom: 1.5). All images are composites, and all scale bars are 10 μm. (E) An example of a connectivity matrix comprised of the sampled input of 200 different Kenyon cell samples. Each row corresponds to a Kenyon cell, and each column corresponds to a glomerulus. Each red dash represents a connection between a Kenyon cell and a given glomerulus, while a yellow dash indicates that a Kenyon cell receives 2 inputs from the same glomerulus.

**Figure 5:**
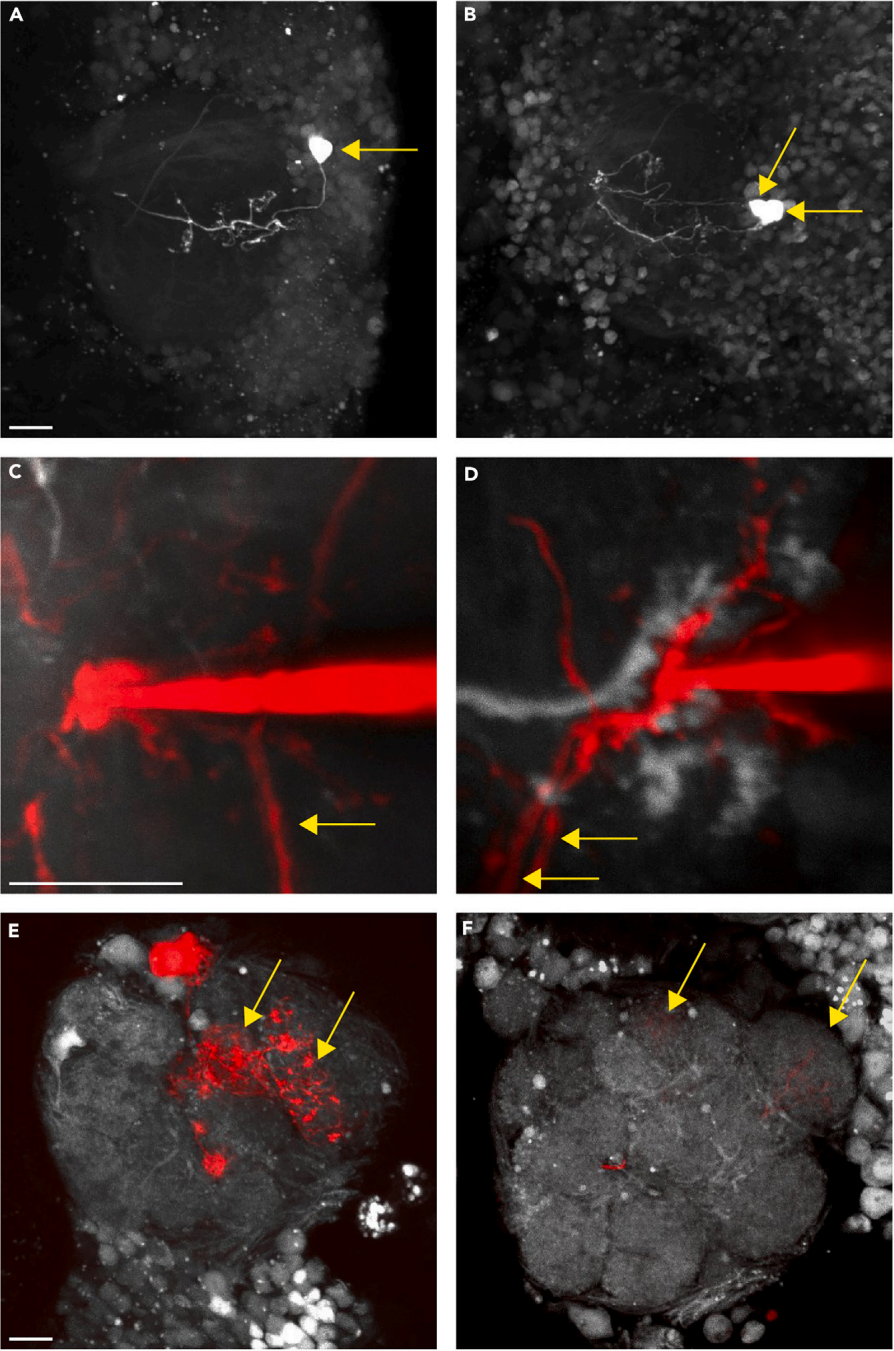
Examples of successful and unsuccessful outcomes (A) The successful outcome of step 2: photo-label a Kenyon cell, in which only one Kenyon cell is labeled (yellow arrow) (digital zoom: 2). (B) An unsuccessful outcome of step 2, wherein two Kenyon cells are photo-labeled (yellow arrows) (digital zoom: 2). (C) A successful outcome of step 3: dye-fill projection neurons, in which only one projection neuron per claw is filled with dye (yellow arrow) (digital zoom: 8). (D) An unsuccessful outcome of step 3, wherein two projection neurons are filled upon dye electroporation into a single claw (yellow arrows) (digital zoom: 8). (E) An antennal lobe with robustly dye-filled glomeruli that are easy to identify (yellow arrows) (digital zoom: 2). (F) An antennal lobe with lightly labeled glomeruli that are difficult to score (yellow arrows) (digital zoom: 2). All images are composites, and all scale bars are 10 μm.

***Note:*** Two image sequences can be acquired: one that includes the somata, and one that is at a larger digital zoom to make it easier to identify the dye-filled glomeruli (suggested digital zooms: 1.5× and 2× respectively) (Figures 4A–4D and 5E).

16. Score the dye-filled glomeruli.
  a. Use one of the publicly available maps of the antennal lobe as a guide for identifying which glomeruli were filled.^4^^,^^7^^,^^8^^,^^9^^,^^10^
  b. Take note of the position of the projection neuron somata, as they can aid in the identification process.
17. Image the mushroom body lobes (Figure 2F).
  a. Acquire a Z-series that includes the αβ, α’β’, and γ mushroom body lobes (suggested digital zoom: 1.5×).

***Note:*** The mushroom body lobes are imaged at this point of the protocol, as opposed to directly after the Kenyon cell is photo-labeled in step 1, because the lobes are more visible from the anterior side of the brain.

18. Determine the identity of the photo-labeled Kenyon cell.
  a. Locate the axons of the photo-labeled Kenyon cell, which should be brighter than the rest of the mushroom body.
  b. Use one of the publicly available maps as a guide to determine which type of lobe its axons project to.^10^^,^^11^
19. Report the data in a connectivity matrix (Figure 4E).
  a. Create a table with one column for each type of glomerulus/projection neuron (there are 51 types) and one row for each Kenyon cell. In our previous studies, we analyzed between 200 and 250 Kenyon cells per genotype.^1^^,^^4^^,^^5^
  b. Additional information for each Kenyon cell sample should be included in the spreadsheet for future analyses. For example:
    i. the type of Kenyon cell;
    ii. the total number of claws a Kenyon cell forms;
    iii. the number of claws that were successfully filled in step 3.

**Pause point:** It is not necessary to score the dye-filled glomeruli and photo-labeled Kenyon cell during or directly after image acquisition. Step 4 sub-steps 16, 18, and 19 can be conducted at a later point or once all samples for a given connectivity matrix are collected.

20. Re-score the connectivity matrix.
  a. Once all samples are collected for a given matrix, it is important to re-score the entire matrix. This step will ensure that the experimenter is confident in the combination of glomeruli that each Kenyon cell sample receives input from; it will also ensure that the experimenter is consistent in their identification of each glomerulus type.
  b. First, re-score each sample (i.e., each row of the matrix) 5 times to reach a consensus.
  c. Next, re-score the matrix 5 times by glomerulus type (i.e., each column of the matrix), comparing all glomeruli of the same type to ensure that identification is consistent across samples.

## Expected outcomes

During the data-collection steps (steps 2–4), the morphology of a Kenyon cell should be visible in both the calyx and lobe of the mushroom body; three or more of the projection neurons connected to the photo-labeled Kenyon cell should be dye-filled; the glomeruli innervated by the dye-filled projection neurons should be visible and identifiable in the antennal lobe.

Figure 2D depicts a successfully photo-labeled Kenyon cell with distinguishable, claw-shaped dendritic terminals.

Figure 3F depicts a sample in which at least three projection neurons are successfully dye-filled.

Figures 4A–4D depicts multiple planes of an antennal lobe showing identifiable, dye-filled glomeruli.

The expected outcome is a connectivity matrix. We have generated such matrices in previous studies and analyzed them to show that antennal lobe to mushroom body connectivity exhibits two qualities. First, the connectivity pattern is random, with a given Kenyon cell receiving input from a set of glomeruli that is stochastic in nature. Second, the connectivity frequencies follow a non-uniform distribution, wherein a select few glomeruli are overrepresented in the dataset, while others are underrepresented.

## Limitations

While our method of mapping mushroom body input is strong in many respects, it is not synonymous with a complete connectome of the mushroom body, and, therefore, might fail to reveal some patterns in connectivity between projection neurons and Kenyon cells that a connectome could reveal. Our method also lacks the resolution to detect minute details, such as active zone number or the number of Kenyon cell claws that envelop a given projection neuron presynaptic bouton.

## Troubleshooting

### Problem 1

The pH and the osmolarity of the saline solution are not within the optimal range (preparation 2, sub-step 4: prepare the saline solution).

### Potential solution

If the pH of the saline is below 7.3, add 20 μL of the 10 M NaOH solution, one drop at a time, and measure the pH again. Repeat until the pH reaches around 7.3. If the pH of the saline is above 7.3, add deionized, filtered water until the pH drops back around 7.3.

### Problem 2

More than 1 Kenyon cell is labeled during the photo-labeling process (step 2: photo-label a Kenyon cell, see Figures 5A and 5B).

### Potential solution

To avoid photo-labeling more than one Kenyon cell at a time, draw a ROI smaller than the original one within the soma and/or use lower laser power during photo-conversion. Another cause of off-target labeling is movement of the brain sample; make sure to securely pin the brain and to check that no movement is occurring before photo-labeling a Kenyon cell.

### Problem 3

The same Kenyon cell type is consistently selected, leading to a dataset in which the other types are underrepresented (step 2: photo-label a Kenyon cell).

### Potential solution

To obtain a dataset with equal representation of each Kenyon cell type, lightly photo-label a mushroom body lobe that corresponds to the desired Kenyon cell type prior to selecting a Kenyon cell to label. This additional step will illuminate the soma of the Kenyon cell type of interest, rendering them slightly brighter than the soma of the other types. Next, select one of the brighter Kenyon cells to robustly photo-label.

***Note:*** If the mushroom body lobe is labeled too robustly, the claws of more than one Kenyon cell will be visible. If this occurs, discard the sample, as proceeding would produce results that are inaccurate.

### Problem 4

More than one projection neuron is labeled during the electroporation of dye into a single Kenyon cell claw (step 3: dye-fill the projection neurons, see Figures 5C and 5D).

### Potential solution

Before electroporating dye into a given claw, verify that the tip of the electrode falls within the claw in all planes, including the z-plane. One way to double check that the electrode is, indeed, within the claw, is to turn down the gain of the red channel; the tip of the electrode is difficult to see once any debris is stuck to it, but it is more visible when the gain is decreased. Another option is to toggle between selecting and deselecting the red channel; this option will make it easy to see the position of the claw relative to that of the electrode to ensure that the claw is selectively targeted. If the shape of the electrode itself is the problem, discard the electrode and replace it. The parameters for pulling electrodes might need to be adjusted or the electrodes might require more fire-polishing to achieve a shape that fits within a claw. Refer to the table in the materials and equipment section that lists the suggested parameters. Off-target labeling can also occur if the voltage that is used to electroporate dye into the claw is too high, a mistake that can be easily prevented by reducing the voltage in the future.

### Problem 5

Debris, such as cell bodies, adhere to the electrode, making it difficult to see the tip of the electrode and fill Kenyon cell claws with precision or at all (step 3: dye-fill the projection neurons).

### Potential solution

This stickiness can occur when the electrode is not fire-polished or is not fire-polished enough. Alternatively, this issue can arise when the brain is incubated in collagenase for too short of a duration or is incubated in collagenase that is too low in concentration. Further dilution of the collagenase can occur if the entire protocol is repeated multiple times in one day, as saline can be transferred with the brain, gradually altering the concentration (step 1: prepare the *Drosophila* brain sample). Another possibility is that the collagenase has expired.

### Problem 6

The dye-filled glomeruli are faint and/or difficult to distinguish from unlabeled glomeruli (step 3: dye-fill the projection neurons and/or step 4: score the dye-filled glomeruli, see Figures 5E and 5F).

### Potential solution

Faint labeling of glomeruli can arise from using dye that is too dilute or, more likely, from not electroporating enough dye into the claw. In some cases, more than one pulse is necessary to adequately dye-fill a projection neuron and its cognate glomerulus. If a bouton does not look robustly labeled directly after electroporation, administer another pulse or two, depending on the appearance of the bouton post-electroporation. However, proceed with caution, as off-target labeling can occur if too many pulses are administered or if any movement of the electrode occurs between pluses. Another potential cause is administering pulses that are too low in voltage to be effective. One solution for weak labeling is to use the fluorescence un-mixing tool while imaging the antennal lobe to enhance the red channel. This solution only works if the dye-filled glomeruli are already somewhat visible. If they are so faint that they are hardly noticeable, the sample cannot serve as a datapoint.

## Resource availability

### Lead contact

Further information and requests for resources and reagents should be directed to and fulfilled by the lead contact, Sophie Caron (sophie.caron@utah.edu).

### Materials availability

This study did not generate new reagents or transgenic lines. The transgenic lines used in this protocol are available upon request.

## Data Availability

All raw data, the connectivity matrices and the code used to analyze these matrices are available on https://github.com/ishanigan/hayashi-et-al-2022. Any additional information required to reanalyze the data reported in this paper is available from the lead contact.
